# Actinic cheilitis: Proposal of a clinical index

**DOI:** 10.4317/medoral.25243

**Published:** 2022-06-05

**Authors:** Cristianne Kalinne Santos Medeiros, Maria Luiza Diniz de Sousa Lopes, Éricka Janine Dantas da Silveira, Kenio Costa Lima, Patrícia Teixeira de Oliveira

**Affiliations:** 1 DDS, MSc, PhD Student, Postgraduate Program in Dental Sciences, Department of Dentistry, Federal University of Rio Grande do Norte, Natal, RN, Brazil; 2 DDS, MSc, PhD, Professor, Department of Dentistry, Federal University of Rio Grande do Norte, Natal, RN, Brazil; 3 DDS, MSc, PhD, Professor, Postgraduate Program in Dental Sciences, Department of Dentistry, Federal University of Rio Grande do Norte, Natal, RN, Brazil

## Abstract

**Background:**

Actinic cheilitis is a potentially malignant lesion most commonly found in the lower lip of individuals with chronic exposure to ultraviolet radiation. The aim of this study was to develop and to test a clinical index that can be used to assess the severity of actinic cheilitis.

**Material and Methods:**

The clinical index of actinic cheilitis was applied to 36 patients. An incisional biopsy was obtained to grade oral epithelial dysplasias following the World Health Organization (WHO) and binary systems, and to evaluate their association with clinical characteristics by Fisher’s exact test (*P*<0.05). The accuracy of the index was evaluated based on sensitivity, specificity, positive and negative predictive values, and receiver operating curve.

**Results:**

The blurring between the border of the lip and the skin was significantly associated with cases without dysplasia/mild epithelial dysplasia (*P*=0.041) and with low risk of malignancy (*P*=0.005). Ulcers and crusts were significantly associated with moderate/severe epithelial dysplasia (*P*=0.002 and *P*=0.012, respectively) and high risk of malignancy (*P*=0.005 and *P*=0.045, respectively). Erosion showed a significant association only with high-risk cases of malignancy (*P*=0.024). The cut-off values of the diagnostic test showing the best performance were 10 for the WHO grading system and 11 for the binary system.

**Conclusions:**

The index cut-offs with the highest accuracy were considered indicators for a biopsy. Erosion, ulceration and crusts were associated with more severe oral epithelial dysplasias.

** Key words:**Actinic cheilitis, solar cheilosis, lip, precancerous conditions, oral diagnosis, epitelial dysplasia.

## Introduction

Actinic cheilitis (AC) is a potentially malignant lesion most commonly found in the lower lip of fair-skinned individuals with chronic exposure to ultraviolet (UV) radiation. If untreated, this condition can progress to lip carcinoma (1,2). Although many studies have examined the association between AC and lip squamous cell carcinoma, the risk of malignant transformation of AC remains uncertain, as reported in the systematic review of Dancyger *et al*. (3) that aimed to evaluate the rate of malignant transformation of AC. However, only one article met the inclusion criteria established by the authors. According to that article, the rate of malignant transformation of AC to lip squamous cell carcinoma is 3.07%. This result highlights the need for more clinical studies on the potential risk of malignant transformation of AC.

The clinical alterations observed in the lip of patients with AC progress slowly during exposure to UV radiation. These alterations include lip atrophy, dryness, desquamation, white lesions, erosion, ulcers, and blurred demarcation between lip and skin, among others (4-7). In general, these alterations occur together and are not homogenous, a fact that makes it difficult for the professional to identify more severe cases and cases that require a biopsy to investigate the progression to lip carcinoma.

Microscopically, the epithelial alterations in AC comprise different degrees of keratinization and epithelial hyperplasia or atrophy, as well as different degrees of epithelial dysplasia. The underlying connective tissue frequently exhibits solar elastosis and an inflammatory infiltrate mainly composed of lymphocytes (8,9). However, previous studies had found no association between clinical characteristics such as white and/or red lesions and the grade of histopathological severity of AC (10,11). This observation compromises the professional’s decision-making when and where to perform a biopsy.

Within this context, the aim of this study was to develop a clinical index for AC (CIAC) that could be used to evaluate a set of clinical characteristics present in patients with AC to provide a severity score for these alterations, identifying cases with an indication for biopsy. In this sense, an auxiliary tool for decision-making and clinical management of these patients.

## Material and Methods

- Study design

This was an observational study conducted in two steps. The first step consisted of the development of a clinical index for assessing the severity of AC, followed by analysis of the accuracy of this index.

- First step: development of the clinical index

First, an exhaustive literature search was performed to identify the clinical characteristics associated with AC for bibliographic validation of these features. The Medline/PubMed, Scopus and Google Scholar databases were searched for articles published in English or Portuguese from 1999 to 2016 using the following keywords: actinic cheilitis, solar cheilosis, lip, and precancerous conditions.

After determination of the clinical characteristics associated with AC, forms containing these features were sent electronically between April and November 2016 to 52 masters and doctors in Stomatology and Oral Pathology with at least two years of experience in clinical care of patients with AC. The instrument contained three questions: 1) the professional was asked to identify, among the listed clinical characteristics (resulting from the literature review), those comprising the clinical presentation of QA; 2) the professional should then indicate which of the characteristic identified by him are present in mild, moderate, or severe AC; 3) If you checked the “Other changes” option, what are they?

The answers to the questionnaires were analyzed and the clinical characteristics indicated as being representative of the clinical presentation of AC by 90% of the experts were listed and the remaining ones were discarded. The tertile of these characteristics was calculated and the values located in the second and third tertiles were selected. Next, each characteristic as mild, moderate or severe was determined for inclusion in the index according to the classification in which they were most frequently cited. Finally, the clinical index containing these characteristics was elaborated by assigning a score according to severity grade, with 1 for mild characteristics of AC, 2 for moderate characteristics, and 3 for severe characteristics. Thus, after application of the clinical index, each patient received a final score corresponding to the sum of points assigned to the clinical presentation of AC to be used as a parameter for clinical decision-making.

- Second step: validation of the clinical index

The second step consisted of a diagnostic study. First, the CIAC was applied to patients with a clinical suspicion of AC attending the Stomatology Service of the Department of Dentistry, UFRN, between May 2017 and November 2019. The study sample was intentional and not probabilistic. Participants older than 18 years of age with a clinical diagnosis of AC were included in this study. Participants with a histopathological diagnosis incompatible with AC were excluded, resulting in a sample of 36 participants.

The participants were examined by a trained and calibrated researcher. Intraexaminer agreement was determined using the weighted kappa test by evaluating 10 participants at an interval of 15 days between applications of the CIAC and was classified as almost perfect agreement (Ƙ = 0,83; *P* < 0.05). During application of the index, the characteristics that were present on the participants’ lips were recorded and scores were then assigned to each characteristic. Finally, these values were summed to obtain a final score and an incisional biopsy was performed in the area showing the highest degree of clinical severity of AC.

Briefly, the specimens were routinely fixed in 10% formaldehyde, embedded in paraffin wax, cut into 5 μm-thick sections, and stained with hematoxylin and eosin. One oral pathologist, who was unaware of the clinical data, carried out the histopathological analysis under a light microscope to classify oral epithelial dysplasias according to the World Health Organization (WHO) grading system published in 2017 and Binary Grading Systems (12).

- Statistical analysis

Data were tabulated and analyzed using the Statistical Package for the Social Sciences (SPSS, version 23.0). First, descriptive analysis of the data was performed. Sensitivity, specificity, positive predictive value (PPV), and negative predictive value (NPV) were calculated to assess diagnostic performance. Receiver operating characteristics (ROC) curves were constructed to assess the sensitivity and specificity of different cut-off values of the clinical index according to the histopathological grading systems used. Fisher’s exact test was used to investigate the association of each individual clinical characteristic with the histopathological grades of oral epithelial dysplasia. The level of statistical significance was set at 5% (*P*<0.05) for all tests.

## Results

- First step: development of the clinical index

For development of the clinical index for assessing AC severity, we first conducted an exhaustive literature search, which resulted in the identification of 35 clinical characteristics associated with AC. Clinical characteristics that represented symptoms (burning and absence of painful symptoms) or that corresponded to the subjective description of a clinical feature (smooth, rough, and irregular surface) were excluded. Repeated clinical characteristics (desquamation/scaly areas; fissures/cracked areas; white/white-greyish plaques) were combined, resulting in 24 characteristics. Only characteristics present in the second and third tertiles of the frequency of citation by dentists were selected, totaling 16 clinical characteristics. The selected clinical characteristics were then classified as mild (score 1), moderate (score 2) or severe (score 3) and based on these results, the CIAC was developed (Table 1).

- Second step: validation of the clinical index

For validation, the clinical index was applied to 36 patients with a diagnosis of AC. This sample predominantly consisted of men (n=29; 80.6%) aged 40 years or older (n=34; 94.4%) with fair skin (n=24; 66.7%), a history of smoking (n=20; 55.6%), and occupational sun exposure (n=24; 66.7%). In addition, most participants used some type of photoprotection such as a cap/hat (n=27; 75%) (Table 2).

**Table 1:**
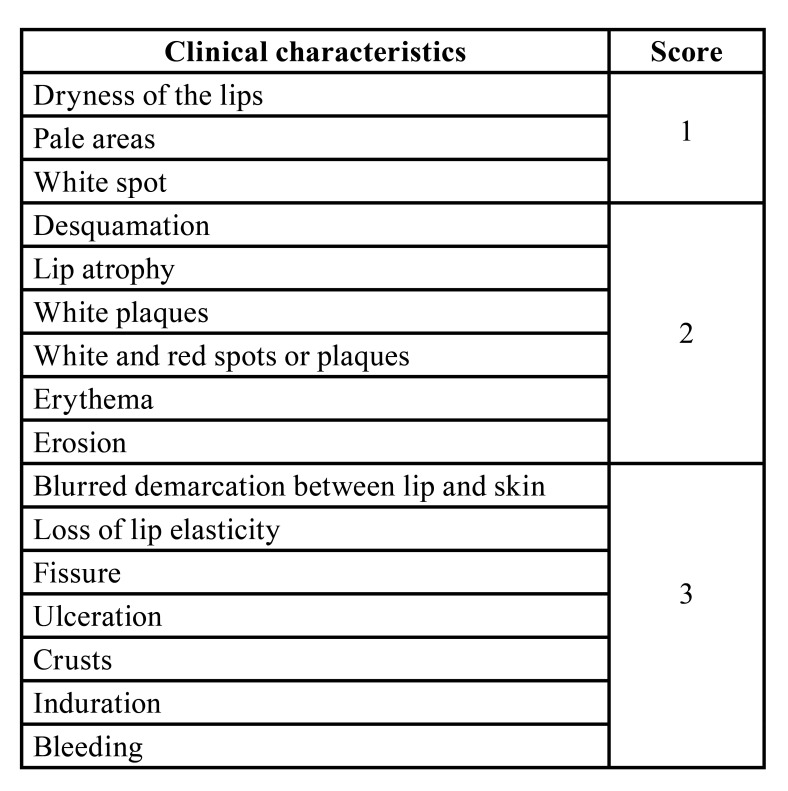
Clinical characteristics selected for the CIAC and their respective clinical severity scores.

**Table 2:**
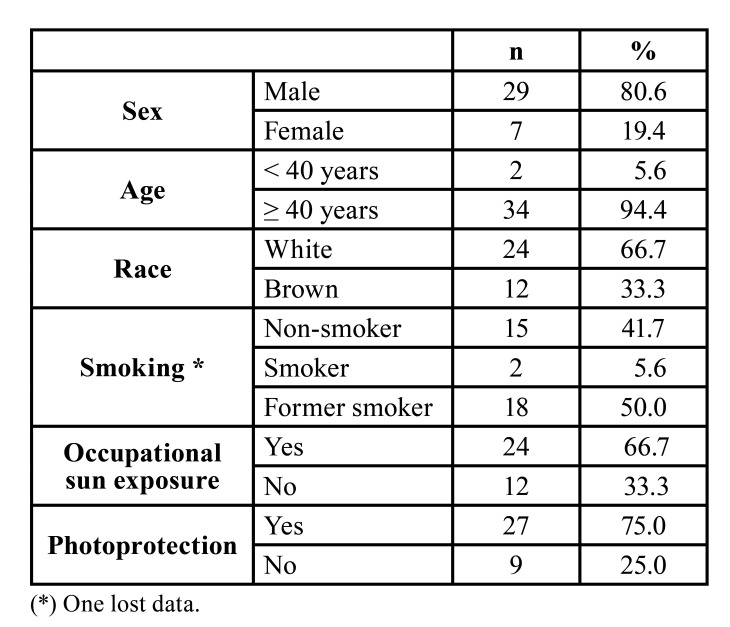
Description of the sample according to demographic data (sex, age, race), smoking, occupational sun exposure, and photoprotection.

**Figure 1:**
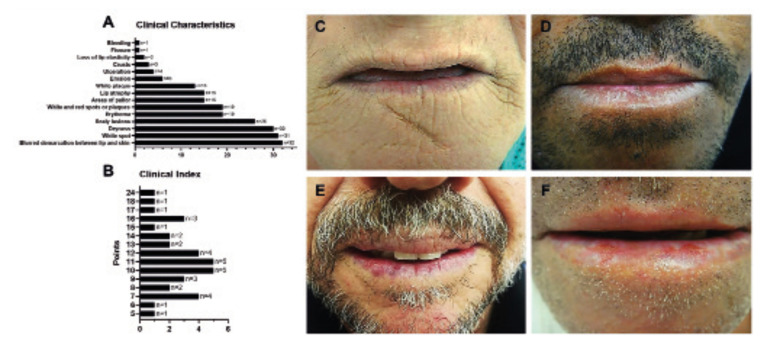
Clinical characteristics of the 36 patients with actinic cheilitis examined. Graphs illustrating the distribution of (a) clinical characteristics and (b) clinical index points of the patients. Representative clinical photographs of the lower lip of the patients showing (c) dryness, white spot, white plaque, erythema, lip atrophy, and blurred demarcation between lip and skin (11 points); (d) dryness, scaly area, white spot, and blurred demarcation between lip and skin (7 points); (e) dryness, white spot, scaly area, white and red spots or plaques, erythema, and blurred demarcation between lip and skin (11 points), and (f) dryness, pale areas, white spot, white and red spots or plaques, erythema, erosion, blurred demarcation between lip and skin, and ulceration (15 points).

With the exception of induration, all characteristics of the clinical index were observed upon physical examination of the patients with AC. The most frequent characteristics were blurred demarcation between lip and skin (n=32; 88.9%), white spots (n=31; 86.1%), dryness (n=30; 83.3%), and desquamation (n= 26; 72.2%) (Fig. 1). Most patients scored 10 (n=5; 13.9%) and 11 points (n=5; 13.9%), followed by 12 (n=4; 11.1%) and 7 points (n=4; 11.1%) after application of the clinical index (Fig. 1).

**Figure 2:**
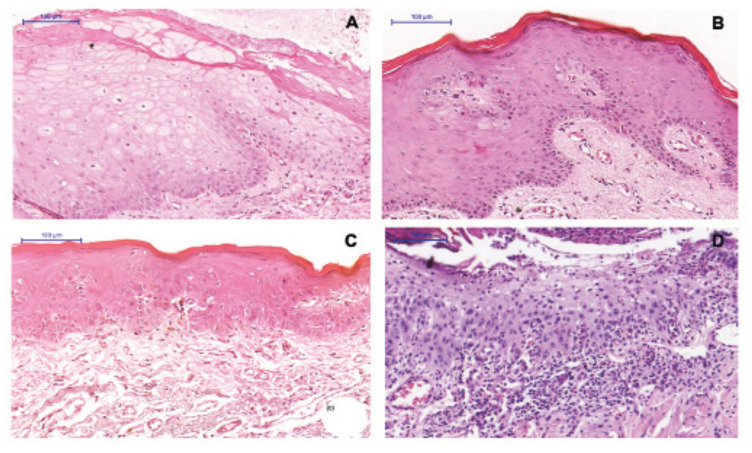
Oral epithelial dysplasias according to the WHO and binary grading systems. (a) Without dysplasia, (b) mild epithelial dysplasia, (c) moderate epithelial dysplasia, (d) severe epithelial dysplasia; (a and b) low risk of malignancy, (c and d) high risk of malignancy (HE; bar =100 μm).

Morphological analysis revealed that 80.6% of the sample had some degree of epithelial dysplasia (Fig. 2).

Using the WHO histological grading system, most cases (n=20; 55.6%) were classified as mild epithelial dysplasia, 6 (16.7%) as moderate dysplasia and 3 (8.3%) as severe dysplasia, while epithelial dysplasia was absent in 7 cases. Using the binary system, 31 cases (86.1%) were classified as low risk of malignancy and 5 (13.9%) as high risk. In view of the low frequency of cases without dysplasia and with severe epithelial dysplasia, the WHO grading system was dichotomized into two groups for the subsequent analyses: cases without dysplasia/mild epithelial dysplasia and cases with moderate/severe epithelial dysplasia.

**Figure 3:**
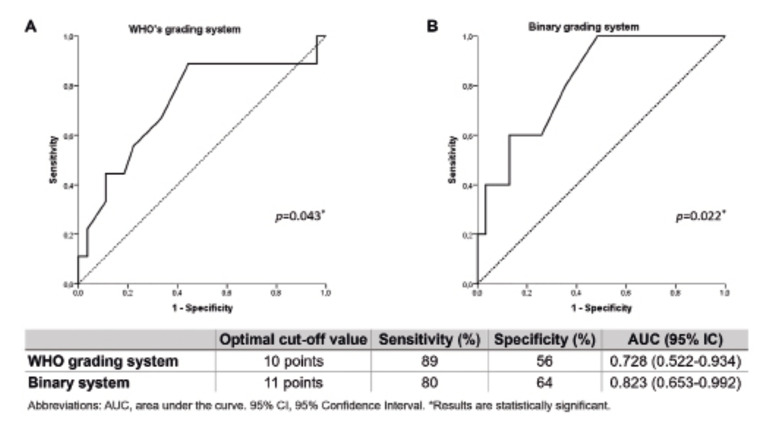
Receiver operating characteristics (ROC) curve for the clinical index used to assess actinic cheilitis (AC) severity. (a) ROC curve constructed for AC patients with moderate/severe dysplasia vs patients without dysplasia/mild dysplasia according to the WHO system; (b) AC patients with low-risk vs high-risk lesions. Optimal cut-off values of the clinical index for each histopathological grading system.

ROC analysis revealed that the optimum cut-off value of the CIAC as clinical diagnostic tool to differentiate among patients with more severe AC was 10 and 11 points (Fig. 3). Detailed information about sensitivity, specificity, PPV, NPV, and accuracy is presented in the Supplementary Material. The area under the curve (AUC) of the CIAC was 0.823 (*P*=0.022) for the binary grading system, while a smaller AUC was obtained for the WHO grading system (0.728, *P*=0.043).

Additionally, association tests were performed between the individual clinical characteristics and the two grading systems used (Table 3, Table 4). Using the WHO grading system, a significant association was observed between the blurred demarcation between lip and skin (*P*=0.041) and cases without dysplasia/mild dysplasia, as well as between the presence of ulceration (*P*=0.002) and crusts (*P*=0.012) and moderate/severe dysplasia. The blurred demarcation between lip and skin (*P*=0.005) was also significantly associated with cases of low risk of malignancy, while erosion (*P*=0,024), ulceration (*P*=0,005) and crusts (*P*=0,045) were associated with a high risk of malignancy by the binary system.

**Table 3:**
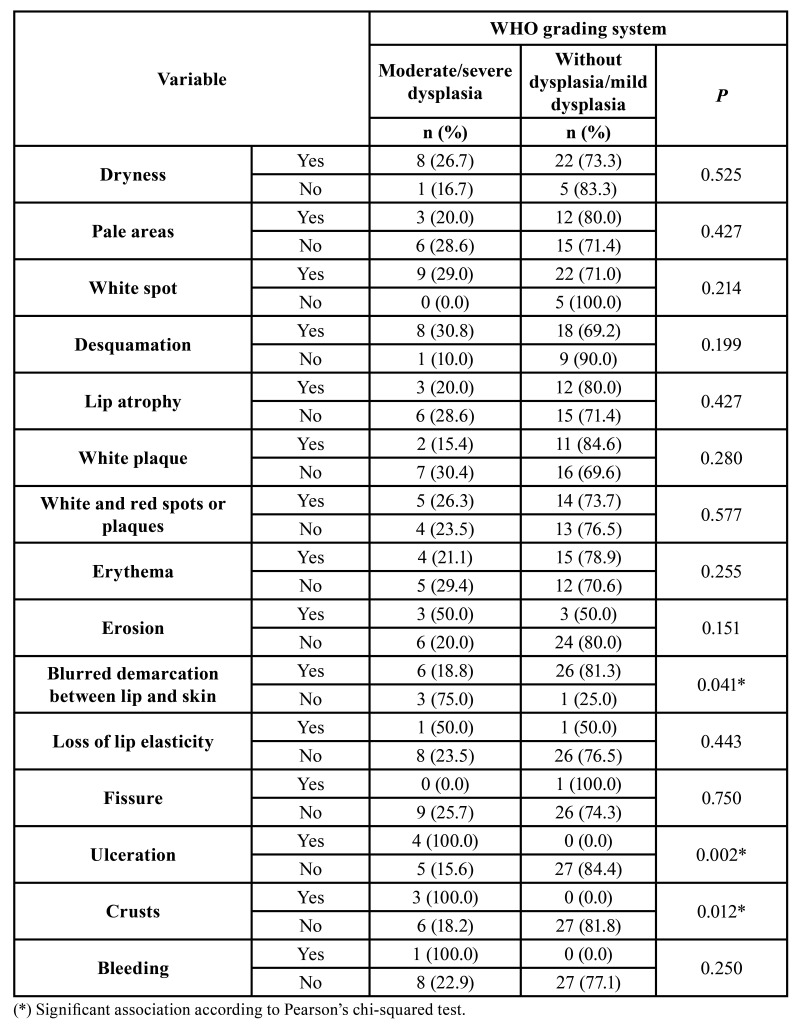
Relative and absolute frequency of the clinical characteristics of actinic cheilitis according to the WHO histopathological grading system (EL-NAGGAR *et al*., 2017).

**Table 4:**
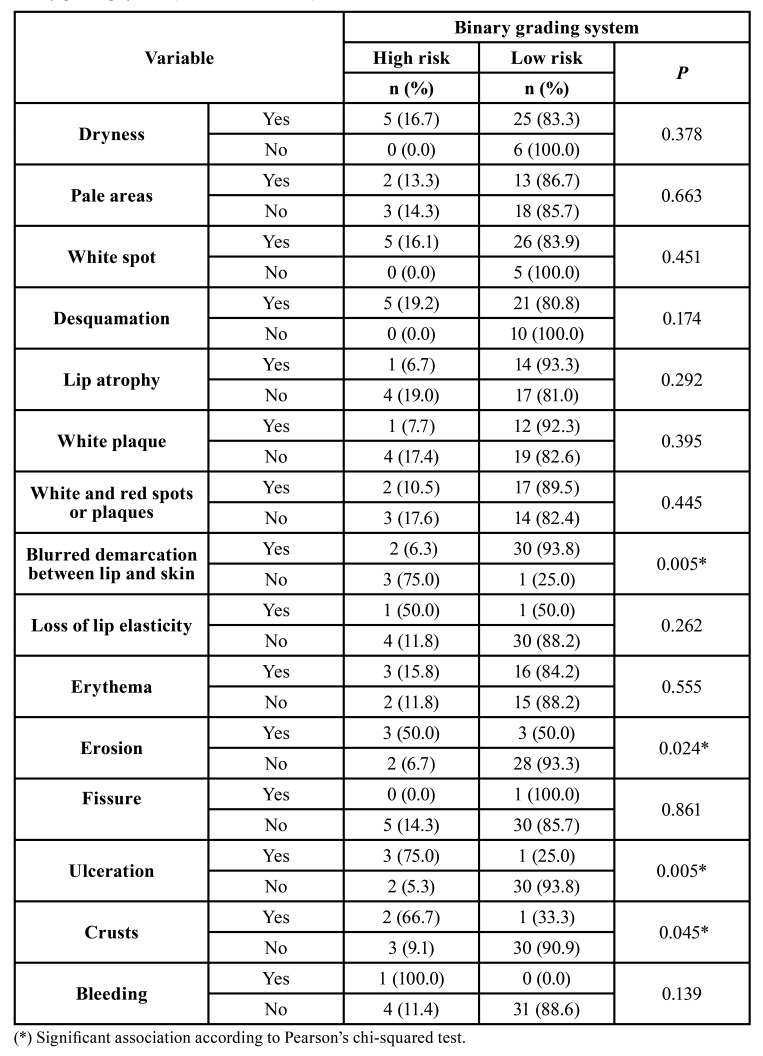
Relative and absolute frequency of the clinical characteristics of actinic cheilitis according to the binary grading system (KUJAN *et al*., 2006).

## Discussion

The clinical management of patients diagnosed with AC is often a challenge for the professional since the clinical presentation of this lesion does not always correspond to its histopathological severity. Thus, procedures that improve diagnostic accuracy are necessary since the treatment and follow-up of these patients are essential for the prevention or early diagnosis of malignant transformation of this lesion.

The profile of the majority of patients with AC of the present sample was consistent with the literature (1,11,13), with a predominance of adult men whose occupational activities involve exposure to solar radiation. White subjects accounted for a significant proportion (66.7%) of our sample and most participants used photoprotective measures such as a cap or hat, similar to the findings of Santos *et al*. (14). Only 5.6% of the participants were smokers, while former smokers accounted for the largest proportion of our sample (50.0%). The latter did not exhibit severe clinical and/or histopathological alterations, although tobacco can act synergistically with UV radiation.

Among the clinical characteristics observed in the CIAC, the blurred demarcation between lip and skin, dryness, desquamation and white spots were the most frequent, in agreement with the data obtained with the questionnaire answered by the experts and the results of other studies (7,15). However, Markopoulos *et al*. (1) found a higher prevalence of red lesions on the lips of patients with AC due to the presence of erosions. In contrast, white lesions, especially white spots, were more common in the present study. This difference might be due to the clinical diagnosis of actinic lesions in early stages, in which the frequency of erosive lesions is lower.

Morphological analysis revealed that most of the present cases had mild epithelial dysplasia (55.6%) according to the WHO grading system. Similar results have been reported in previous studies (2,9,10,16). In contrast, Cavalcante *et al*. (15) found a high prevalence of AC cases with severe epithelial dysplasia in their study. These differences between studies might be related to the clinical presentation of AC at the time of biopsy since some professionals decide to perform surgical procedures only when erosive and/or ulcerated lesions are present.

As observed for the WHO grading system, the results obtained based on the binary system also showed a larger number of mild alterations in the epithelium; 86.1% of the cases analyzed were classified as low risk of malignancy, similar to the studies of Lopes *et al*. (11) and Câmara *et al*. (9). In contrast, in the study of Pilati *et al*. (17), 63.8% of the cases of AC were classified histopathologically as high risk of malignancy.

Individual analysis of the clinical characteristics of Ac showed a statistically significant association between the blurred demarcation between lip and skin and cases without dysplasia/mild dysplasia and low risk of malignancy. Only four of the 36 participants did not exhibit blurred demarcation between lip and skin (focal or diffuse). The latter is a relatively common characteristic in patients with AC.

Severe clinical characteristics such as ulcers and crusts, as well as erosion classified as moderate in the present study, are frequent in patients chronically exposed to the sun. In this study, these characteristics were associated with cases of greater histopathological severity. However, the degree of clinical alterations in AC may often not be related to the extent of damage found in the epithelium and connective tissue (15,16).

Poitevin *et al*. (18) proposed a clinical score for AC (Grade I starting from dryness of vermillion to ulcers representing Grade IV) and evaluated its reproducibility. The authors concluded that the clinical score for AC was easily applicable and had good reproducibility; however, the authors did not perform a morphological study of the AC cases included in this analysis. To our knowledge, no studies in the literature have validated a clinical index that could be used to measure both the grade of clinical severity of AC and the need for a biopsy.

In the present study, the most accurate cut-off value to identify more severe AC was 10 for the WHO grading system of oral epithelial dysplasias and 11 for the binary system. Although cut-off values of 5 and 7 for the WHO system and 5, 7, 8 and 9 for the binary system also showed 100% sensitivity, their specificities were very low for the two histopathological grading systems, resulting a large number of false-positive individuals. Additionally, the predictive value of a test is not only determined by sensitivity and specificity but also by the prevalence of the disease in the population. If the disease prevalence is very low, the PPV tends to be low and classifies many individuals as false-positive even if sensitivity and specificity were high. Thus, the low PPV percentages of the cut-off values tested in the present study are probably due to the lower frequency of AC cases with a higher histopathological severity grade.

Ideally, diagnostic instruments should have high sensitivity and specificity. However, in practice, available tests do not always provide these results. This can also be applied to the present study since none of the cut-off values analyzed provided excellent results. We therefore evaluated a set of indicators for the diagnostic test that could provide the most efficient cut-off value for the CIAC.

The CIAC was found to be a promising diagnostic tool, showing good reproducibility, easy applicability, rapidity, and low cost. However, further prospective studies involving a larger patient cohort to validate the reproducibility of the index are necessary.

Demographic data of the sample analyzed reinforce the profile of patients with AC already established in the literature. Clinical characteristics such as erosion, ulceration and crusts were significantly associated with a higher histopathological severity grade of the lesion. The CIAC cut-off values of 10 and 11 using the WHO and binary grading systems, respectively, showed the best accuracy and these values should be used for biopsy indication. The use of CIAC may contribute to reduction of subjectivity of the decision-making process in the clinical management of AC patients.
